# Invited Commentary: The Clinical Significance of Lymph Node Ratio and Ki-67 Expression in Papillary Thyroid Cancer

**DOI:** 10.1007/s00268-021-06087-3

**Published:** 2021-03-26

**Authors:** Marcin Barczyński

**Affiliations:** Department of Endocrine Surgery, Third Chair of General Surgery, Medical College, Jagiellonian University, Kraków, Poland

Thyroid cancer is one of the most common endocrine malignancies, with increasing incidence all over the world. Papillary thyroid cancer (PTC) accounts for 85% of all thyroid cancers and is generally an indolent tumor, treated effectively with surgery, radioactive iodine (RAI) and thyroid-stimulating hormone suppressive therapy.

This retrospective Swedish study comprised of 165 patients with PTC operated on at Karolinska University Hospital in 2009–2011 with total thyroidectomy, elective central lymph nodes dissection (CLND) and selective lateral neck dissection (LND) [1]. Such a management approach was in accordance with the Swedish national guidelines prevailing the years of the study period, which in turn were based on American Thyroid Association (ATA) 2009 recommendations. The Ki-67 proliferation index ≥ 3% was found to be correlated to lymph node (LN) metastasis and tumor recurrence in PTC (sensitivity 50%, specificity 80%). The lymph node ration (LNR) ≥ 21% was associated with tumor recurrence regardless the anatomical site of cervical LN metastases (sensitivity 89%, specificity 70%). Hence, authors concluded that these findings indicate that Ki-67 and LNR better reflect the malignant behavior of advanced PTC and constitute useful prognostic indicators in addition to the well-established conventional TNM classification.

Since the ATA's guidelines for the management of these disorders were revised in 2015, many aspects of thyroid cancer management remain not uniformly accepted [2]. In general, there is a raising awareness that de-escalation of overtreatment in low-risk PTC is safe with no long-term disease-specific survival decrement. However, European panelists suggested modifications to approximately one-third of ATA 2015 recommendations [3]. Current controversies in the field include active surveillance as an option for selected patients with low-risk PTC, lobectomy as an initial therapy for PTC, the proper use of preoperative neck imaging to optimize the completeness of the initial surgical procedure, indications for prophylactic CLND, the selective use RAI therapy as remnant ablation, adjuvant treatment or treatment of known persistent/recurrent disease.

According to the recent ATA 2015 guidelines, prophylactic CLND clearance of clinically negative central LNs is recommended in patients with advanced primary tumors (T3 or T4) and/or positive lateral nodes (N1b). Prophylactic CLND can be abandoned in patients with T1 and T2 tumors and clinically negative central LNs. In this study, authors have shown that LN metastases were associated with T stage and occurred mainly in T3 and T4 tumors. This observation supports the notion that prophylactic CLND can be refrained in patients with T1 and T2 tumors. Hence, the message presented in this Swedish study indicating that LNR might be useful in assessment of patients with T3 and T4 tumors is consistent with recent ATA 2015 recommendations. Some other earlier studies in the field provided matching evidence that central LN macro- but not micro-metastases together with positive to negative LNR ≥ 0.3 (which typically occurs in larger tumors) represent the strongest independent prognostic factors for the PTC recurrence in the lateral neck [4].

Unfortunately, biologically aggressive variants of PTC like hobnail, tall cell, columnar and solid were not taken into consideration in the analysis of this study cohort [5]. As shown by outcomes of recent study reported by Nath et al. mortality risk was substantially increased in patients with hobnail variant of PTC harboring BRAF mutation and BRAF mutation associated with TP53 and/or PIK3CA mutations. The detection of these multiple mutations in a gene panel appears to allow the identification of a subset of more aggressive tumors within the group and to bear information that should be useful for prognostic stratification of these patients including the planning of adjuvant therapy [6]. It becomes clear that future improvements in the management of PTC should include identification of biologically aggressive variants of PTC based on their molecular signatures recognized in fine-needle aspiration residual washout before tailoring surgical approach. As thyroid cancer management moves toward a much more risk adapted approach to personalized management recommendations, clinicians and patients must balance the risks and benefits of the potential management options to arrive at a management plan that is optimized based on both patient preferences and the experience of the local disease management team. Personalized treatment with estimates based on classical prognostic factors and enhanced by a novel molecular profiling has an increasing potential to improve further the current management of PTC.
